# Improved Power Control Using Optimal Adjustable Coefficients for Three-Phase Photovoltaic Inverter under Unbalanced Grid Voltage

**DOI:** 10.1155/2014/538520

**Published:** 2014-08-27

**Authors:** Qianggang Wang, Niancheng Zhou, Xiaoxuan Lou, Xu Chen

**Affiliations:** ^1^State Key Laboratory of Power Transmission Equipment and System Safety and New Technology, Chongqing University, Chongqing 400044, China; ^2^Department of Electrical Engineering, University of Wisconsin Milwaukee, Milwaukee, WI 53211, USA; ^3^Guangdong Key Laboratory of Clean Energy Technology, South China University of Technology, Guangzhou 510640, China

## Abstract

Unbalanced grid faults will lead to several drawbacks in the output power quality of photovoltaic generation (PV) converters, such as power fluctuation, current amplitude swell, and a large quantity of harmonics. The aim of this paper is to propose a flexible AC current generation method by selecting coefficients to overcome these problems in an optimal way. Three coefficients are brought in to tune the output current reference within the required limits of the power quality (the current harmonic distortion, the AC current peak, the power fluctuation, and the DC voltage fluctuation). Through the optimization algorithm, the coefficients can be determined aiming to generate the minimum integrated amplitudes of the active and reactive power references with the constraints of the inverter current and DC voltage fluctuation. Dead-beat controller is utilized to track the optimal current reference in a short period. The method has been verified in PSCAD/EMTDC software.

## 1. Introduction

Nowadays, photovoltaic generation (PV) has played a vital role in distributed generation systems for its easy installation and absence of fuel cost [1, 2]. Grid-connected PV generation can be divided into centralized generation and distributed generation. For the last few years, the government has supported the distributed grid-connected PV generation (whose installed capacity is less than 6MW) by the demonstration project and subsidy policy, and some international technical standards have been proclaimed for grid-connected PV station. In these technical standards, it is clearly stipulated that each PV generation has to be connected to the grid constantly and provide reactive power support under unbalanced grid fault [3–5].

However, the unbalanced grid voltage sag will make the DC voltage and output powers of PV inverters fluctuate heavily. At the same time, the output current will rise and contain a large amount of harmonics. The PV generation system has to quit operation once either of the DC voltage fluctuation, the output current peak, or distortion exceeds the limit values [6]. Hence, it is not only necessary to analyze the operation characteristics of PV systems, but also important to research on the control strategy of PV systems under unbalanced voltage sag.

According to the safe operation restrain of power converters, a dual synchronous coordinate current control method has been proposed to keep the active power in a constant value under unbalanced voltage [7]. Then the algorithm of the current reference has been improved to maintain the DC voltage constant if the filtering inductance is assumed to absorb the power fluctuation [8, 9]. Keeping the DC voltage and power unchanged under unbalanced voltage sag will make the output current rise. Based on the limited current peak that inverters can bear, [10] has put forward a solution that the exchange power between the inverter and the grid should be reduced to limit the output current amplitude. Consider the influence caused by the injected current of inverters to power grid; the control schemes are designed based on the characteristics of power fluctuation and harmonics [11, 12]. And a control strategy in which the amplitudes of active and reactive power fluctuation are continuously adjustable has been proposed in [13]. In allusion to the grid-connected PV generation system, two adjustable coefficients (*α* and *β*) have been added into the algorithm of current reference which are determined by the constraints of current harmonics and power fluctuation [6]. Another coefficient adjustment method of current reference was proposed to reduce the peak current in [14]. However, the present coefficient adjustment algorithms of current references only consider either one of the limit facts [15] (the current peak, harmonics distortion, and DC voltage). They have not presented the feasible regions of the adjustment coefficients for all the limits, and the way of choosing the optimal coefficients has not been mentioned. In a word, according to the present references, PV generation systems cannot satisfy all the former three operation indices at the same time.

Under some practical operation conditions, especially in the night or cloudy daytime, the output active power of the PV generation is usually lower than the rated capacity of the inverter, and the PV generation has a proper capability to adjust reactive power [16, 17]. It is usually assumed that, under unbalanced grid fault, the PV system operates under unity power factor, which means the output reactive power is usually zero. However, it is necessary to utilize PV converters to provide reactive power support to the grid under unbalanced voltage sag. Hence, PV converter control should be designed considering the constraints of the current peak, the harmonics, and the DC voltage limits, and then the low-voltage ride-through ability of PV generations under unbalanced voltage can be significantly improved.

The operation features of a PV generation system under unbalanced grid voltage have been demonstrated by the authors in [15]. This paper is an extension of our preliminary survey in [15] and focuses on the control design of three-phase minidistributed PV inverters. Taking the constraints including the active and reactive power fluctuation, the current peak, and the harmonics into account, this paper has proposed an improved control strategy in which three adjustment coefficients (*α*, *β*, and *γ*) are brought in to tune the current reference. Then the paper has presented adjustable expressions of some typical operating parameters, including the total harmonics distortion (THD), the phase-to-ground current peak, and the power and DC voltage fluctuation. The impacts on control performance caused by the current adjustment coefficients and the feasible region of the coefficients have been discussed. Considering the constraints of the inverter current and the DC voltage fluctuation, the optimal model of the output current reference is established, aiming to generate the minimum integrated amplitudes of the active and reactive power. Then the process of choosing the coefficients of power control is proposed. Finally, the feasibility of the proposed control strategy is verified with PSCAD/EMTDC simulation software.

## 2. Improved Power Control of PV Inverter under Unbalanced Grid Voltage

**Figure 1:**
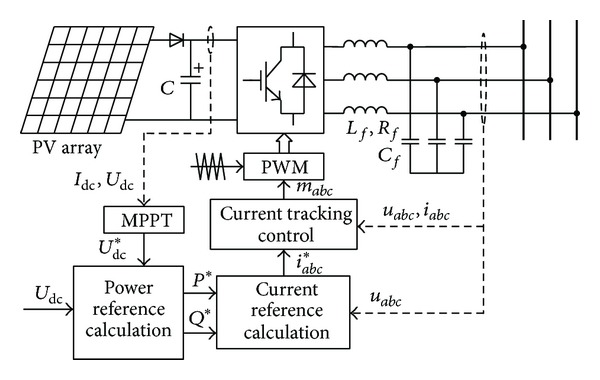
Grid-connected PV generation system structure.

The structure of a three-phase PV generation system, which consists of PV array, power converters, and controllers, is shown in Figure 1. The voltage of the DC capacitor should be regulated properly, so that the PV array will be able to work at the maximum power point (MPP) under different light intensity and ambient temperature. As is shown in Figure 1, the active power reference P* is generated from the error between the capacitor voltage *U*_dc_ and the reference voltage *U*_dc_* generated by the maximum power point tracking (MPPT) module. The reactive power reference Q* can be obtained by the reactive power generation strategy.

In addition, combining the above with grid voltage *u*_*abc*_, three-phase current reference value *i*_abc_* can be generated, and, moreover, the PV inverter power control can be realized through the current tracking loop. There are many studies on photovoltaic MPPT and DC voltage control at present [18, 19]. The paper will mainly discuss the power control strategy under unbalanced grid faults. The distribution network is either generally neutral grounded or arc suppression coil grounding [11], so that there are no zero-sequence voltage and current in the three-phase three-wire system. The terminal voltage and current of PV inverter only contain positive- and negative-sequence components which are expressed as

(1) u(t)|a,b,c=2U+cos⁡[ωt−(m−1)2π3+φu+]+2U−cos⁡[ωt+(m−1)2π3+φu−],

(2) i(t)|a,b,c=2I+cos⁡[ωt−(m−1)2π3+φi+]+2I−cos⁡[ωt+(m−1)2π3+φi−],

where *m* = 1,2, 3 means the three phase *a* *b* *c*, while *U* ^+^, *U* ^−^ and *φ*_*u*_^+^, *φ*_*u*_^−^ are the amplitudes and phase angles of positive- and negative-sequence voltage. Likewise, *I* ^+^, *I* ^−^ and *φ*_*i*_^+^, *φ*_*i*_^−^ are the amplitudes and phase angles of positive- and negative-sequence current, respectively. *ω* = 2*πf* (*f* = 50 Hz) is the grid frequency.

The output current and voltage of the PV generation can be written in space forms as **u** = **u** ^+^ + **u** ^−^ and **i** = **i** ^+^ + **i** ^−^, where **u** ^+^ = [*u*_*a*_^+^,*u*_*b*_^+^,*u*_*c*_^+^]^*T*^, **u** ^−^ = [*u*_*a*_^−^,*u*_*b*_^−^,*u*_*c*_^−^]^*T*^, **i** ^+^ = [*i*_*a*_^+^,*i*_*b*_^+^,*i*_*c*_^+^]^*T*^, and **u** ^−^ = [*i*_*a*_^−^,*i*_*b*_^−^,*i*_*c*_^−^]^*T*^. Then the calculation of three-phase instantaneous active power can be described as the dot product of the current vector and the voltage vector:

(3) p=u·i=u+·i++u−·i−+u+·i−+u−·i+=3[U+I+cos⁡⁡(φu+−φi+)+U−I−cos⁡⁡(φu−−φi−)  +U+I−cos⁡⁡(2ωt+φu++φi−)  +U−I+cos⁡(2ωt+φu−+φi+)].

In order to simplify the calculation, the voltage vector can be calculated as $u_{⊥}={\left[u_{⊥a},u_{⊥b},u_{⊥c}\right]}^{T}={\left[u_{b}-u_{c},u_{c}-u_{a},u_{a}-u_{b}\right]}^{T}/\sqrt{3}$ which is an orthogonal vector of **u**. Vector **u**_⊥_ can be expressed as

(4) u⊥(t)|a,b,c=2U+sin[ωt−(m−1)2π3+φu+]+2U−sin[ωt+(m−1)2π3+φu−],

where **u**_⊥_ = **u**_⊥_^+^ + **u**_⊥_^−^ is also combined with positive-and negative-sequences. The instantaneous reactive power of three-phase photovoltaic inverter can be expressed as

(5) q=|u×i|=u⊥·i=u⊥+·i++u⊥−·i−+u⊥+·i−+u⊥−·i+=3[U+I+sin⁡(φu+−φi+)+U−I−sin⁡(φu−−φi−)  +U+I−sin(2ωt+φu++φi−)  +U−I+sin(2ωt+φu−+φi+)].

Under an unbalanced grid fault, a reverse cross of the positive- and negative-sequence components of the voltage and current will result in fluctuations of the active and reactive power in a three-phase system. The PV generation should also be fully used to output reactive power on the premise that the PV inverters will not quit operation under unbalanced voltage sag. Based on (1) and (4), the module values of **u**_⊥_ and **u** are equal and it is obvious that |**u** ^+^+**u** ^−^|^2^ = |**u**_⊥_^+^+**u**_⊥_^−^|^2^. In order to maintain the active and reactive power of PV inverter under unbalanced faults, the current reference can be obtained from (3) and (5):

(6) i∗=P∗(u++u−)|u++u−|2+Q∗(u⊥++u⊥−)|u⊥++u⊥−|2=P∗(u++u−)|u+|2+2u+u−+|u−|2.

If the current reference presented above is used in tracking control, the power of PV inverter will be able to track the given value without double-frequency fluctuation. However, the harmonic distortion of output current may be out of limit, which may force the PV inverter to quit operation. Therefore, based on expression (6), three coefficients (*α*, *β*, and *γ*) are brought in and the output current reference of PV inverter under unbalanced faults can be written as

(7) $$\begin{array}{c} i^{*}=\frac{P^{*}\left(u^{+}+\alpha u^{-}\right)+Q^{*}\left(u_{⊥}^{+}+\gamma \alpha u_{⊥}^{-}\right)}{{\left|u^{+}\right|}^{2}+\left(1+\alpha \right)\beta u^{+}u^{-}+\alpha {\left|u^{-}\right|}^{2}}. \end{array}$$

Then the harmonics and the amplitude of the output current can be reduced while the fluctuation of active and reactive power will increase slightly by means of choosing proper values of the coefficients *α*, *γ*, and *β* (*α*, *γ* ∈ [−1,1] and *β* ∈ [0, 1]) in (7).

## 3. Performance Analysis of Improved Power Control

### 3.1. Current Harmonic Distortion of PV Generation

Suppose the ratio of negative-sequence voltage to positive-sequence voltage is *U* ^−^/*U* ^+^ = *n*, and based on (7), (1), and (4), we can get the instantaneous values of the three-phase current:

(8) i∗(t)|a,b,c=23[1+β(1+α)ncos⁡(2ωt)+αn2]U+ ·{P∗[cos⁡⁡(ωt−(m−1)2π3)    +αncos⁡(ωt+(m−1)2π3)]  +Q∗[sin(ωt−(m−1)2π3)     +γαnsin(ωt+(m−1)2π3)]}.

Take the current of phase A in (8) as an example; the root-mean-square (RMS) values of the fundamental current *I*_1rms_ and the full-wave current *I*_rms_ are

(9) I1rms=1T[∫0Tia∗(t)cos⁡⁡(ωt)dt]2+[∫0Tia∗(t)sin⁡(ωt)dt]2≈2S∗3U+C(1+B+A),Irms=1T∫0T[ia∗(t)]2dt≈2S∗A6U+C(1+B+A)(1+B−C),

where *T* = 1/*f*, S* = [*P* ^∗2^(1+*αn*)^2^+*Q* ^∗2^(1+*γ* *αn*)^2^]^1/2^, *A* = *β*(1 + *α*)*n*, *B* = *αn* ^2^, and *C* = [(1+*B*)^2^−*A* ^2^]^1/2^. The RMS values of the fundamental-frequency current and the full-frequency current are basically the same when *β* = 0. The deviation between the values of these two currents will increase as *β* increases. The double-frequency component in (8) is the immediate cause of the current harmonics of inverters. From (9), the current THD of PV generation can be derived as

(10) $$\begin{array}{c} \text{THD}=\sqrt{\frac{I_{\text{rms}}^{2}}{I_{1\text{rms}}^{2}}-1}=\sqrt{\frac{A^{2}}{2C\left(1+B-C\right)}-1}. \end{array}$$

**Figure 2:**
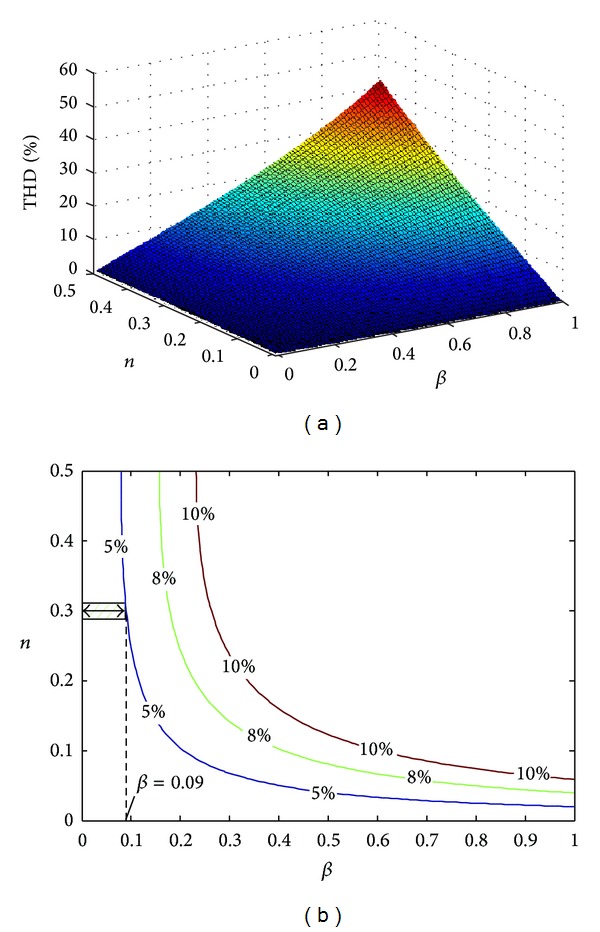
Three-dimensional mesh and contour lines of total current harmonic distortion. (a) Three-dimensional mesh. (b) Contour lines.

Figure 2 has shown the changing trend of the current harmonics distortion with the increasing of coefficients *n* and *β* (when P* = 0.8 pu, Q* = 0.6 pu, *γ* = 1, and *α* = 0.5). When an unbalanced grid fault of *n* = 0.3 happens, the current THD will exceed the international regulation which is 5% if *β* > 0.09. It is obvious that the coefficient *β* has a very small range to choose from. The current harmonics injected into the grid under unbalanced voltage can be reduced by reducing *β*. In order to keep the current harmonics within the specified limit, in this paper, it is assumed that *β* = 0 so that the PV generation system can bear a big negative-sequence voltage.

### 3.2. Phase Current Peak Value of PV Generation

**Figure 3:**
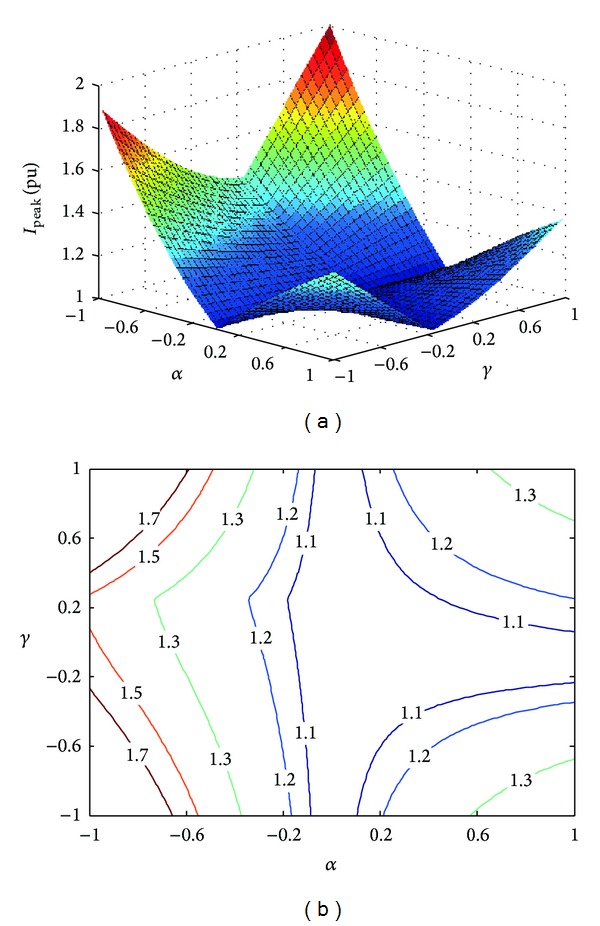
Three-dimensional mesh and contour lines of peak current. (a) Three-dimensional mesh. (b) Contour lines.

The output phase current peak value (*I*_peak_ = max⁡{*i*_a_*, *i*_b_*, *i*_c_*}) can be adjusted by tuning the coefficients *α* and *γ*. Set P* = 0.8 pu, Q* = 0.6 pu, *β* = 0, and *n* = 0.5; we can obtain the three-dimensional mesh and contour lines of the peak current. As is shown in Figure 3, there are four local maximum values at the points where *α* = ±1 and *γ* = ±1. *I*_peak_ is close to the rated current peak when *α* = 0, but there will be a large power fluctuation.

The contour lines when the current peak values are equal to 1.1, 1.2, 1.3, 1.5, and 1.7 pu are shown in Figure 3(b). The feasible regions of coefficients *α* and *γ*, which are encircled by the contour lines, will expand with the increasing phase current peak values that the PV system can sustain. The coefficient point (*α*, *γ*) which corresponds to unbalanced grid fault of *n* = 0.5 is inside its *I*_peak_ contour line region. The phase current peak value of the PV generation will be smaller than the value corresponding to the grid fault of *n* = 0.5. Once any phase current of the PV inverters exceeds the limit, the protection will trip to isolate the PV system from the grid.

### 3.3. Active and Reactive Power Fluctuation of PV Generation

The output current of a PV system in formula (8) (in this case we set *β* to be 0) will only contain the fundamental component. Substitute *β* = 0 into (3) and (5); we can obtain the instantaneous active and reactive powers as

(11) p=P∗[1+(1+α)ncos⁡(2ωt)1+αn2]+Q∗(1−γα)nsin(2ωt)1+αn2=P∗+p~,

(12) q=Q∗[1+(1+γα)ncos⁡(2ωt)1+αn2]+P∗(α−1)nsin(2ωt)1+αn2=Q∗+q~,

where $\tilde{p}$ and $\tilde{q}$ are the double-frequency fluctuation components of the active and reactive powers.

**Figure 4:**
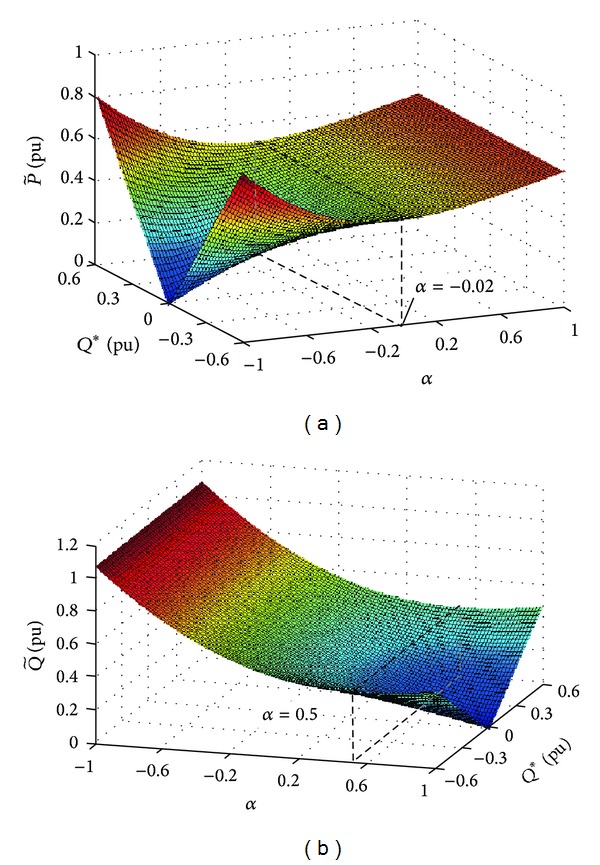
Three-dimensional mesh of active and reactive power fluctuation magnitudes. (a) Amplitudes of active power fluctuation. (b) Amplitudes of reactive power fluctuation.

Figure 4 has shown the changing trend of the power fluctuation with *α* and Q*. If we set Q* = 0, $\tilde{p}$ will increase and $\tilde{q}$ will decrease as *α* increases. If the PV system injects reactive power into the grid under unbalanced fault, the reactive power reference Q* will cause fluctuation in both $\tilde{p}$ and $\tilde{q}$. In Figure 4, if Q* = ±0.6 pu (as is shown in the dashed lines), the amplitudes of both $\tilde{p}$ and $\tilde{q}$ will get to the minimal values when *α* = −0.02 or 0.5.

### 3.4. DC Voltage Fluctuation of PV Generation

**Figure 5:**
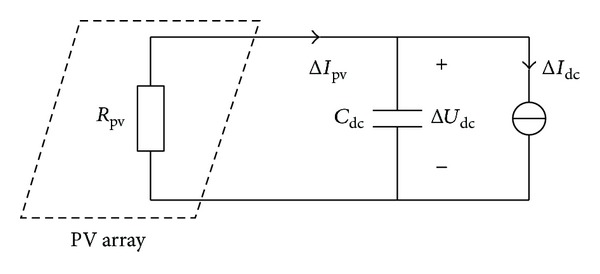
Equivalent circuit for DC voltage fluctuation.

Active power fluctuation will result in slight fluctuations in the DC voltage. Assume that the illumination and the temperature of the PV array remain unchanged and the PV array operates steadily at the maximum power point; the equivalent internal resistance of the PV array can be written as *R*_pv_ = *U*_dc_/*I*_dc_ [20]_,_ as is shown in Figure 5, where *U*_dc_ and *I*_dc_ are the voltage and current of the maximum power point before grid fluctuation occurs.

In Figure 5, the DC current fluctuation caused by the active power fluctuation is approximately ${\tilde{i}}_{\text{dc}}=\tilde{p}/U_{\text{dc}}$, and the double-frequency component of the DC current can be obtained from (11). The amplitude of the DC voltage fluctuation ${\tilde{U}}_{\text{dc}}$ can be expressed as

(13) $$\begin{array}{c} {\tilde{U}}_{\text{dc}}=\frac{nR_{\text{pv}}\sqrt{{\left[P^{*}\left(1+\alpha \right)\right]}^{2}+{\left[Q^{*}\left(1-\gamma \alpha \right)\right]}^{2}}}{U_{\text{dc}}\left(1+\alpha n^{2}\right)\sqrt{1+{\left(2\omega CR_{\text{pv}}\right)}^{2}}}. \end{array}$$

**Figure 6:**
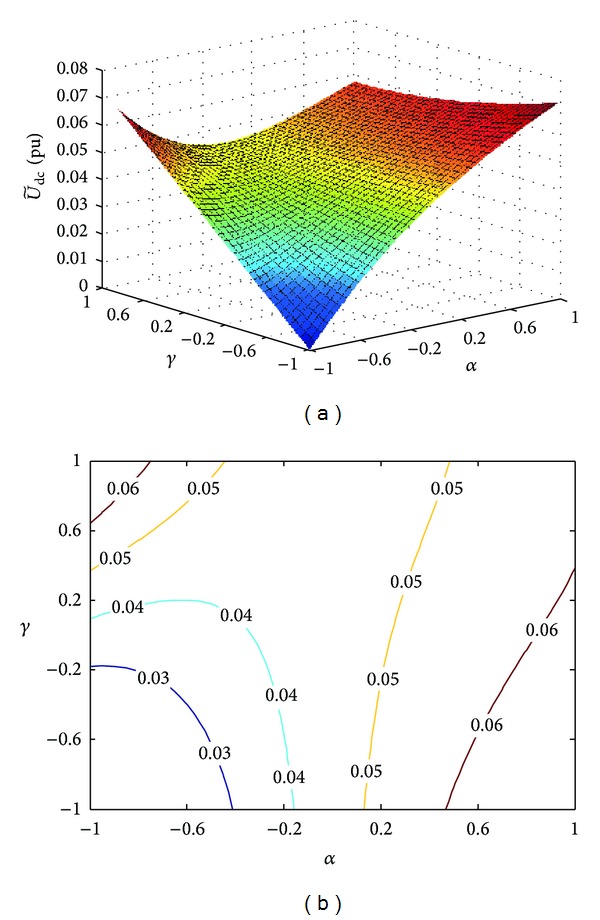
Three-dimensional mesh and contour lines of DC voltage fluctuation. (a) Three-dimensional mesh. (b) Contour lines.

The changing trend of the amplitude of DC voltage fluctuation with *α* and *γ* is shown in Figure 6, where P* = 0.8 pu, Q* = 0.6 pu, *U*_dc_ = 800 V, *I*_dc_ = 5 A, *C* = 1800 *μ*F, and *n* = 0.5. Similarly, the given limit value of the DC voltage fluctuation will also determine the feasible regions of the coefficients *α* and *γ*. Once any of the indices (the current harmonic distortion, the peak value of phase current, and the amplitude of DC voltage fluctuation) exceeds the limit, the PV generation system will be forced to quit operation, so that it is necessary to choose the coefficients *α* and *γ* properly.

## 4. Optimal Coefficients Selection and Improved Power Control Implementation

### 4.1. Optimal Coefficients Selection

In order to reduce the power fluctuation and keep the voltage and current fluctuation below the required limits at the same time, this paper has linearly weighted the amplitudes of power fluctuation ($\tilde{p},\tilde{q}$) and built the optimal selection model of the coefficients *α* and *γ*, aiming at getting the minimum synthesis power fluctuation with the constraints of the peak phase current limit and the DC voltage fluctuation limit. The objective function can be expressed as

(14) min⁡f=n1+αn2{ωp[P∗(1+α)]2+[Q∗(1−γα)]2 +ωq[P∗(α−1)]2+[Q∗(1+γα)]2},

where *ω*_*p*_ and *ω*_*q*_ are the weighting coefficients of the active and reactive power fluctuation and *ω*_*p*_ + *ω*_*q*_ = 1. The constraint conditions are

(15) max⁡{23(1+αn2)U+   ×{P∗[cos⁡(ωt−(m−1)2π3)       +αncos⁡(ωt+(m−1)2π3)]     +Q∗[sin⁡(ωt−(m−1)2π3)       +γαnsin(ωt+(m−1)2π3)]}}<Imax⁡,m=1,2,3,

(16) $$\begin{array}{c} 0<\frac{nR_{\text{pv}}\sqrt{{\left[P^{*}\left(1+\alpha \right)\right]}^{2}+{\left[Q^{*}\left(1-\gamma \alpha \right)\right]}^{2}}}{U_{\text{dc}}\left(1+\alpha n^{2}\right)\sqrt{1+{\left(2\omega CR_{\text{pv}}\right)}^{2}}}<{\tilde{U}}_{\text{dcmax}}, \end{array}$$

where *I*_max⁡_ and ${\tilde{U}}_{\text{dcmax}}$ are the maximum limit values of AC phase current peak and the DC voltage fluctuation. Set the rated capacity of the PV system as *S*_*n*_; the reactive power reference should be selected within the region [−(*S*_*n*_^2^−*P* ^∗2^)^1/2^, (*S*_*n*_^2^−*P* ^∗2^)^1/2^]. If there is no intersection between the feasible regions of the coefficients *α* and *γ* determined by (15) and (16), the reactive power reference Q* should be corrected through iteration. This paper has built an optimal model of the coefficients *α* and *γ* with the constrained cyclic coordinate descent method. The procedure of the coefficients selection of power control can be summarized.

*Step  1.* Record P*, *U*_dc_, and *I*_dc_ before voltage sag and input *U* ^+^, *n*, and Q* after the disturbance, and set *k* = 1, *η*_*k*_ = 1, Δ*η* = 0.01, and *Q*_*k*_^∗′^ = *η*_*k*_ Q*.

*Step  2.* Judge whether all of the points in the plane (*α*, *γ*) satisfy (15) and (16) with the same equal interval method: Δ*α* = Δ*γ* = 0.05. 

*Step  3.* If there is a point (*α*, *γ*) not satisfying the condition, set *k* = *k* + 1, *η*_*k*_ = *η*_*k*−1_ − Δ*η*, and *Q*_*k*_^∗′^ = *η*_*k*_ Q*, and go to Step 2. Otherwise, set *X*_0_ = {(*α*, *γ*)∣*α* + *γ* = min⁡(*α* + *γ*)} and go to Step 4.

*Step  4.* Choose *X*_0_ as the starting point: Q* = *Q*_*k*_^∗′^; set the search step Δ*s* = 0.001, the precision *ε* = 0.002, and *j* = 1. Then go to Step 5. 

*Step  5.* Search using a fixed step towards the *α* according to the direction *X*_*j*_^(1,*l*)^ = *X*_0_ + *l*Δ*s* × (*l*, 0), *l* = 1,2, 3,…, and calculate *f*[*X*_*j*_^(1,*l*)^] by (14). 

*Step  6.* If *f*[*X*_*j*_^(1,*l*)^] increases or *X*_*j*_^(1,*l*)^ does not satisfy (15) or (16) when *l* = *l*_max⁡_, stop increasing *l* and *X*_*j*_^(1)^ = *X*_*j*_^(1,*l*−1)^. 

*Step  7.* Search towards *γ* with *X*_*j*_^(2,*l*)^ = *X*_*j*_^(1)^ + *l*Δ*s* × (0, 1), and *X*_*j*_^(2)^ can be found, which is recorded as the optimal point of the *j*th search, namely *X*_*j*_ = *X*_*j*_^(2)^.

*Step  8.* If |*X*_*j*_ − *X*_*j*−1_ | >*ε*, set *j* = *j* + 1, and go to Step 5. Otherwise, output the optimal coefficients *α* and *γ*.

### 4.2. Improved Power Control Implementation

**Figure 7:**
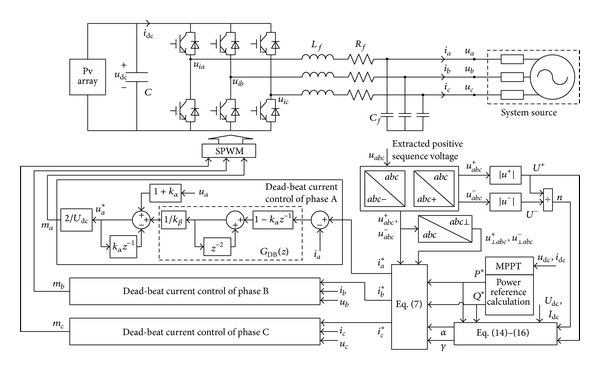
Block diagram for improved power control of photovoltaic generation.

The improved control structure of PV system is shown in Figure 7. The positive- and negative-sequence voltage components (*u*_*abc*_^+^ and *u*_*abc*_^−^) of the PV inverters under unbalanced voltage can be extracted by second-order generalized integrators (SOGI) [11]. From the expressions [*u*_⊥*a*_^+^,*u*_⊥*b*_^+^,*u*_⊥*c*_^+^]^*T*^ = ${\left[u_{b}^{+}-u_{c}^{+},u_{c}^{+}-u_{a}^{+},u_{a}^{+}-u_{b}^{+}\right]}^{T}/\sqrt{3}$ and ${\left[u_{⊥a}^{-},u_{⊥b}^{-},u_{⊥c}^{-}\right]}^{T}={\left[u_{b}^{-}-u_{c}^{-},u_{c}^{-}-u_{a}^{-},u_{a}^{-}-u_{b}^{-}\right]}^{T}/\sqrt{3}$, the positive and negative components of the orthogonal voltages *u*_⊥*abc*_^+^ and *u*_⊥*abc*_^−^ can be calculated. Set the power references P*, Q* and the three-phase unbalance factor *n* as inputs; the optimal coefficients *α* and *γ* can be calculated based on the optimal model in (14) and (16). The three-phase current reference *i*_abc_* of the dead-beat current inner loop can be deduced from (7) where *k*_*α*_ and *k*_*β*_ are the current control coefficients and *z* ^−1^ is the unit delay of a discrete system.

The inner current loop is based on the discrete state equation considering the delay of sampling and controlling, and it can control the inverters at the next sampling period. In Figure 7, the three-phase dead-beat current controllers are the same and adjusting signal is generated by the current error through the control function *G*_DB_(*z*). The control coefficients *k*_*α*_ and *k*_*β*_ in the current inner loop can be determined by the sampling period *T*_*s*_ and the parameters *L*_*f*_, *R*_*f*_, and *C*_*f*_ of filtering circuit [21].

## 5. Simulation Results

A PSCAD/EMTDC simulation model of 2 kW PV generation system in Figure 7 is implemented to illustrate the effectiveness of the proposed method. Comparing the reference DC voltage in MPPT with the actual DC voltage, the active power reference can be obtained through the PI controller and the low pass filter (which is used to eliminate the power fluctuation). The discrete unit delay *z* ^−1^ is replaced by exp⁡(−*sT*_*s*_) in complex frequency domain. The system parameters are listed in Table 1 and based on typical data supplied in [11, 14].

### 5.1. Characteristics Analysis of Current Harmonic Distortion

**Figure 8:**
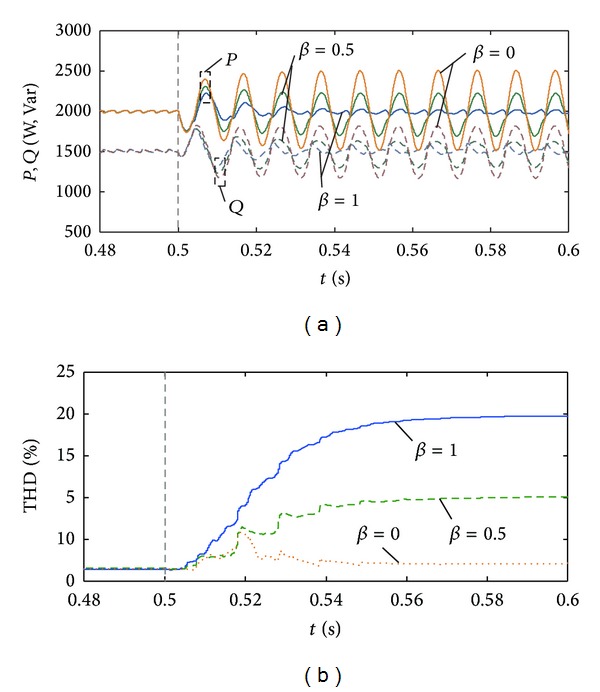
Output powers and current harmonics of photovoltaic generation for different *β* coefficients. (a) Active and reactive power when *n* = 0.2. (b) Total harmonic distortion when *n* = 0.2.

Consider this case: the PV's active and reactive power under normal operation conditions are 2 kW and 1.5 kVar. Negative-sequence voltage sag of *n* = 0.2 appears at *t* = 0.5 s. Set the power control coefficients (*α*, *γ*) = (1,1). In Figure 8, the curves of power fluctuation and current THD of the PV system are under different coefficient *β*. As is shown in the graph, as *β* increases, the current THD increases while the active and reactive fluctuation decrease.

**Figure 9:**
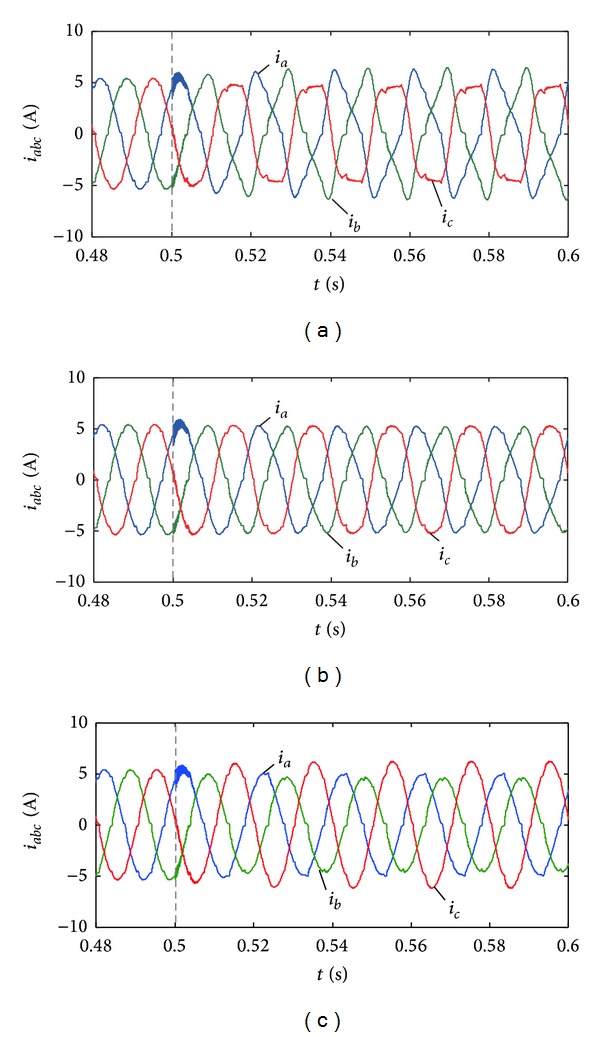
Output currents of photovoltaic generation for different *β* coefficients. (a) *β* = 1, *n* = 0.2. (b) *β* = 0.5, *n* = 0.2. (c) *β* = 0, *n* = 0.2.

The current THDs are 19.72%, 10.05%, and 2.02% when *β* = 1, 0.5, and 0 in Figure 8(b). The calculation results of expression (10) are 20.41%, 9.75%, and 0 which are basically the same as the simulation results. In Figure 9, the instantaneous three-phase current curves are under three conditions. The current distortion after grid disturbance will have the biggest value when *β* = 1, and the current peak value of phases *a*, *b* will rise apparently. When *β* = 0, the output current distortion is small but the current peak value of phase *c* will rise. Hence, it is necessary to choose proper control coefficients (*α*, *γ*) to decrease the phase current peak value below the specified limit.

### 5.2. Sensitivity Analysis of Adjustable Coefficients

**Figure 10:**
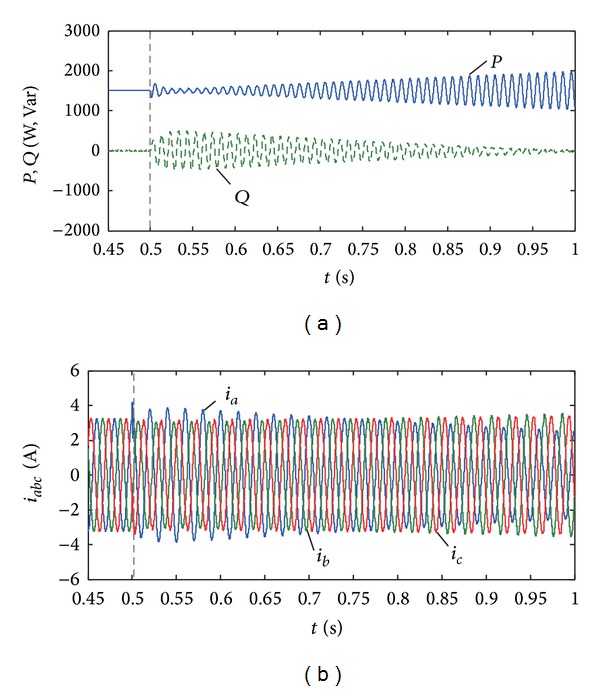
Sensitivity analysis of coefficient *α* for power control. (a) Active and reactive power when *n* = 0.2. (b) Output current when *n* = 0.2.

Suppose the active and reactive powers to be 1.5 kW and 0 Var; the condition of grid unbalanced voltage is the same as the previous case and the PV control coefficient is *γ* = 1 after *t* = 0.5 s.  Figure 10 shows the changing processes of the PV output power and current as the coefficient *α* increases from −1 to 1 with a slope of 4 pu/s. When the output reactive power of the PV system is 0, the active power fluctuation will increase and the reactive power fluctuation will decrease as the coefficient *α* increases, which agrees with the calculation results in Figure 4.

**Figure 11:**
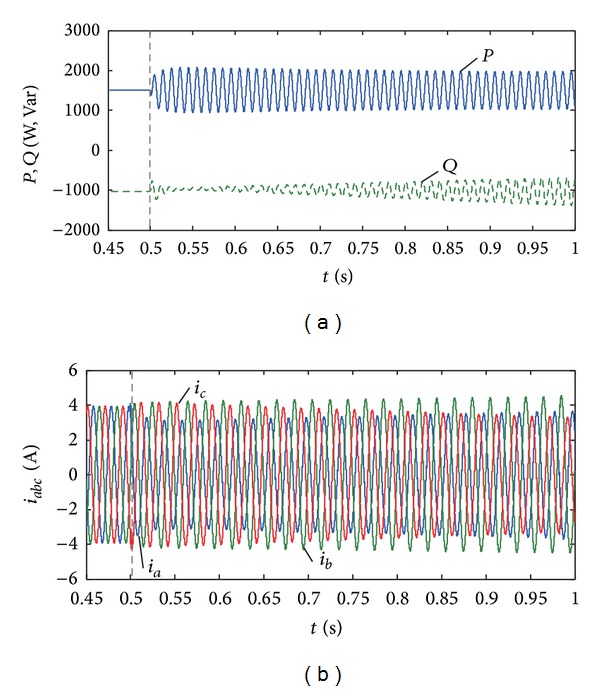
Sensitivity analysis of coefficient *γ* for power control. (a) Active and reactive power when *n* = 0.2. (b) Output current when *n* = 0.2.

Set the active and reactive powers to be, respectively, 1.5 kW and −1 kVar.  Figure 11 shows the changing process of the PV output power and current when the coefficients *α* = 1 and *γ* increase generally from −1 to 1. In this case, the changing coefficient *γ* will have little effect on the active power fluctuation, and the reactive power fluctuation will decrease as *γ* increases. From Figures 10 and 11, the output power fluctuation of PV system can be flexibly controlled by adjusting the control coefficients (*α*, *γ*).

### 5.3. Improved Power Control with Optimal Coefficients

**Figure 12:**
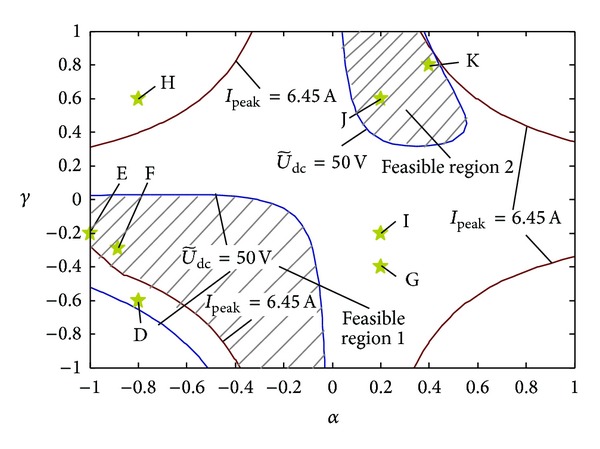
Feasible region for power control coefficients of photovoltaic generation.

In the optimal coefficients power control model, we set *ω*_*p*_ = 0.8 and *ω*_*q*_ = 0.2 and set the active and reactive powers to be 1.5 kW and 2 kVar, respectively. The same as before, a negative-sequence voltage sag of *n* = 0.2 occurs after *t* = 0.5 s, and the optimal coefficients can be calculated by the proposed method as (*α*, *γ*) = (−0.884, −0.290) which is presented by the point F in Figure 12.  Table 2 shows the calculation and simulation results of power fluctuation, DC voltage fluctuation, and current peak values which correspond to different coefficient points from group D to group K in Figure 2. It is obvious that using the optimal coefficient group F, there will be the lowest comprehensive power fluctuation (both active and reactive fluctuation) which is the objective function *f* in (14) and THD in the output current, and the DC fluctuation will be within the limit.

According to the simulation and calculation results of all the indices for different coefficients in Table 2, the maximum relative errors of the current peak, the DC voltage fluctuation, and the power fluctuation (including active, reactive, and comprehensive fluctuation) are 3.38%, 7.89%, and 8.72%, respectively. The relative errors for all the indices are within ±10%, which proves the correctness of the analytic expressions derived in Section 2. The shadow areas, which are encircled by the contour lines of Δ*U*_dcmax_ = 50 V and *I*_peak_ = 6.45 A in Figure 12, are the feasible regions (1 and 2) of control coefficients. The coefficient groups D to K are also marked in Figure 12.

From Table 2 and Figure 12, it is known that for group D, only the phase current peak is beyond the limit; for groups I and G, the current peak is within the limit, while the DC voltage fluctuation exceeds the limit. However, both the current peak and the DC voltage fluctuation are out of limit in group H. In Figure 12, all the indices for E, F, J, and K are in the feasible region.

**Figure 13:**
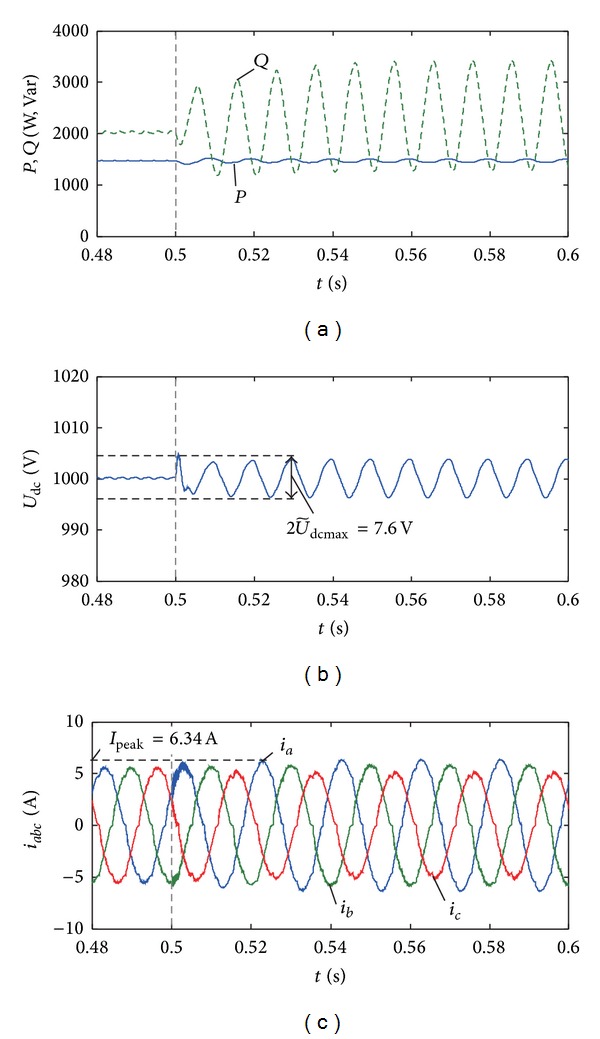
Operating characteristics for optimal control coefficients. (a) Active and reactive power when *n* = 0.2. (b) DC voltage when *n* = 0.2. (c) Output current when *n* = 0.2.

The curves of the output power, the DC voltage, and the three-phase current of PV system under the optimal control coefficients which is in the feasible region 1 are shown in Figure 13. According to the results with coefficient groups J and K in Table 2, the objective function *f* (the comprehensive power fluctuation) in the feasible region 2 has a local minimum value which is still higher than the one with coefficient group F in the feasible region 1. The optimal coefficients used in Figure 13 can achieve the global minimum comprehensive power fluctuation under unbalanced voltage; meanwhile, all the constraints (the current THD, the current peak, and the DC voltage fluctuation) can be satisfied.

## 6. Conclusion

This paper has dug into the flexible coefficients control strategy and proposed an optimal coefficients selection method focusing on the reactive power demand of the grid under unbalanced voltage. The output current can be determined automatically in an optimal way under different specified constraints of power quality. By testing the simulation model of PV generation system based on the dead-beat current controller, the result has proved that the proposed control strategy can restrict the output current THD within the 5% limit. Choosing the coefficients by using the coefficients optimization model can realize a flexible control towards active and reactive power regulation of a PV system, while the values of the phase current peak and the DC voltage fluctuation can meet the constraints at the same time.

## Figures and Tables

**Table 1:** System parameters.

Parameters	Values
*U*_*n*_/V	220
*f*/Hz	50
*L*_*fabc*_/mH	12
*R*_*fabc*_/Ω	0.4
*C*_*fabc*_/*μ*H	0.7
*C*/*μ*H	1800
Short-circuit level of system source/MVA	5
*U*_dc*n*_/V	1000
*f*_*c*_/kHz	16
*T*_*s*_/*μ*s	31.25
*S*_*n*_/kVA	2.5
*I*_max⁡_/A	6.45
${\tilde{U}}_{\text{dcmax}}$/V	50
*X*/*R* ratio of system source	10

**Table 2:** Comparison between calculation and simulation results for different power control coefficients.

Coefficient group (*α*, *γ*)	Calculation value				
	*I*_peak_/A	${\tilde{U}}_{\text{dc}}$/V	$\tilde{p}$/W	$\tilde{q}$/Var	*f*/VA
**D** (−0.8, −0.6)	**6.73**	42.2	385.2	1329.3	574.0
**E** (−1, −0.2)	6.36	9.1	83.3	976.3	261.9
**F** (−0.884, −0.290)	**6.30**	**4.1**	**37.4**	**1024.7**	**234.9**
**G** (0.2, −0.4)	5.61	**69.5**	634.0	359.9	579.2
**H** (−0.8, 0.6)	**6.99**	**132.4**	1208.2	675.1	1101.6
**I** (0.2, −0.2)	5.49	**63.9**	582.6	409.6	548.0
**J** (0.2, 0.6)	5.72	45.2	412.5	633.7	**456.7**
**K** (0.4, 0.8)	6.30	46.9	427.8	915.0	525.2

Coefficient group (*α*, *γ*)	Simulation value				
	*I*_peak_/A	${\tilde{U}}_{\text{dc}}$/V	$\tilde{p}$/W	$\tilde{q}$/Var	*f*/VA

**D** (−0.8, −0.6)	**6.51**	41.5	377.5	1321.5	566.3
**E** (−1, −0.2)	6.35	9.8	89.6	981.8	268.0
**F** (−0.884, −0.290)	**6.34**	**3.8**	**34.4**	**1034.5**	**234.4**
**G** (0.2, −0.4)	5.59	**67.9**	641.3	360.5	585.1
**H** (−0.8, 0.6)	**6.83**	**132.2**	1206.1	672.9	1099.5
**I** (0.2, −0.2)	5.56	**61.4**	577.6	413.7	544.8
**J** (0.2, 0.6)	5.75	43.6	407.2	638.4	**453.4**
**K** (0.4, 0.8)	6.31	45.4	423.2	909.6	520.5
